# Designing resilient decentralized energy systems: The importance of modeling extreme events and long-duration power outages

**DOI:** 10.1016/j.isci.2021.103630

**Published:** 2021-12-11

**Authors:** Ryan Hanna, Jeffrey Marqusee

**Affiliations:** 1 Center for Energy Research, University of California San Diego, La Jolla, CA 92093, USA; 2 Deep Decarbonization Initiative, University of California San Diego, La Jolla, CA 92093, USA; 3 National Renewable Energy Laboratory, Golden, CO 80401, USA

**Keywords:** Energy policy, Energy resources, Energy systems

## Abstract

Mitigating and adapting to climate change requires decarbonizing electricity while ensuring resilience of supply, since a warming planet will lead to greater extremes in weather and, plausibly, in power outages. Although it is well known that long-duration outages severely impact economies, such outages are usually not well characterized or modeled in grid infrastructure planning tools. Here, we bring together data and modeling techniques and show how they can be used to characterize and model long-duration outages. We illustrate how to integrate outages in planning tools for one promising mode of resilient energy supply—microgrids. Failing to treat these extremes in models can lead to microgrid designs (1) that do not realize their full value of resilience, since models do not see the benefits of protecting against extremes, and (2) that appear reliable on paper yet do not actually protect against extremes. Although utilities record power interruptions, lack of access to that data is hindering research on resilience; making datasets available publicly would substantially aid efforts to improve grid planning tools.

## Graphical abstract

**Figure:**
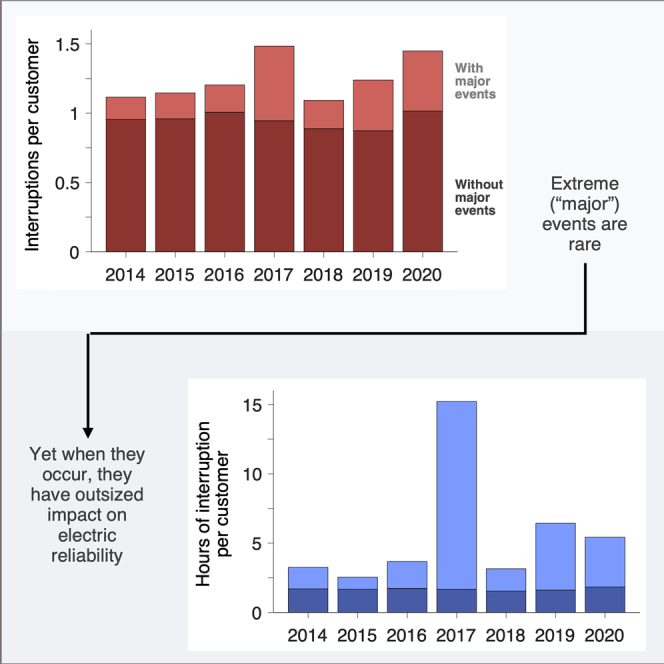

## Introduction

The electric power system is on the cusp of two revolutions. The first is decarbonization—the transition to carbon-free supplies of electricity (National Academy of Sciences, 2021a). At the same time, these new carbon-free energy resources are downsizing and increasingly being deployed as decentralized supplies at the “grid edge” (National Academy of Sciences, 2021b). The transition to decarbonized, decentralized electricity is creating enormous opportunities for customer-sited energy resources like solar photovoltaics, batteries and electric vehicles (Hanna and Victor, 2021). It is also creating opportunities for new grid architectures, like microgrids, that can keep energy from these sources reliable (Gholami et al., 2016) (National Academy of Sciences, 2017). The opportunities are substantial: since 2014 US microgrid deployments have increased year over year, boosted by government support following Superstorm Sandy. The year 2019 alone saw nearly 550 new installations, a record high (Wood Mackenzie, 2021a, 2021b). With technology costs falling rapidly (Way et al., 2021), capital markets, too, are backing the project: since 2018 US private equity firms have committed over $1 billion for new microgrids, including a $500 million investment in 2021, the largest ever (Greentech Media, 2021) (UtilityDive, 2021).

Microgrids promise to address what are, arguably, the two principal challenges facing the electric power industry: the need to decarbonize while maintaining or even increasing today's levels of reliability and resilience (Parhizi et al., 2015). By utilizing renewable and other resources, microgrids can shield customers from outages by disconnecting from the grid during disruptions and operating autonomously, reducing the number and duration of interruptions that customers experience (Hussain et al., 2019). Hence microgrids have a “value of resilience”—they avoid the economic, health, safety, and security losses caused by power outages (Anderson et al., 2019) that cost the US economy billions each year (LaCommare et al., 2018) and are projected to cost $1.5–3.4 trillion through mid-century (Larsen et al., 2018). The massive size of the market for resilience—estimated in one recent study as a $500 billion resilience investment gap across US utilities (Bruzgul and Weisenfeld, 2021)—helps explain the recent substantial investments in microgrids as well as utility spending on system hardening and other resilience measures (Georgia Power, 2019) (ConEd, 2021).

Efforts to deploy resilient infrastructure in the real world, however, face numerous challenges. First, unlike reliability, resilience is still an emerging concept in power system planning (Panteli and Mancarella, 2017), and there are no agreed-upon frameworks for measuring it (Zamuda et al., 2019). Second, even if a framework were standardized, calculations of reliability and resilience are methodologically demanding. Investments increase reliability and resilience, but increases are nonlinear and eventually they diminish (Billinton and Allan, 1996). Knowing how much improvement the next dollar of investment buys requires detailed characterization of the many types of outages, particularly extremes that are rare but impactful, known to occur. Third, extremes are growing in number and severity (Larsen et al., 2020) (Zamuda et al., 2018) and are outpacing the planning tools used to design infrastructure, as others have recently highlighted (Brockway and Dunn, 2020) (McCollum et al., 2020) (Otto et al., 2020). Microgrids planning tools—our focus—help determine the set of investments, like generation sources and energy storage, that maximize a microgrid's financial benefits over its lifetime. But models make “correct” investment decisions only insofar as they know how costs are incurred and where benefits can be derived; they must therefore know about the full nature of disruptions, from momentary to sustained to long-duration outages, that cause economic losses.

This paper focuses on these two latter challenges—the need to update planning tools to include better and more thorough calculations of reliability and resilience. Addressing these challenges will require better characterization of long-duration outages and, in turn, we argue, new research efforts on two fronts: data analysis and modeling.

First, fresh analysis of raw power outage data is needed to generate reliability functions (i.e., probability distributions) for the likelihood and duration of different types of disruptions. Ideally, one would construct distributions by aggregating data from a wide cross-section of individual outage events and customer interruptions, as in Hossein Afsharinejad et al. (2021) and Dunn et al. (2019). Although utilities record these granular data, they are not typically available for public use. (Utilities, with the help of regulators and industry, could do much more to release granular, anonymized interruption data for public use.) Since those data are not commonly available, a next-best approach is to use aggregated interruption data that electric utilities report as annual reliability indices. We present these data and discuss how they can be worked into distinct classes of outages that can be included in models.

On the second front, complementary efforts, similar to Bennett et al. (2021), are needed to assimilate outage distributions in planning tools. This will require expanding the suite of existing tools or building new tools altogether. Tools today are typically built as linear programs, which are computationally efficient but limited in their ability to capture the diversity of events that impair reliability. Reliability is a nonlinear function of investment, so linear models must make a number of simplifying linearizations. One limitation, for example, is the small number of outage events that they can efficiently analyze. With changes, however, or new approaches altogether, a fuller spectrum of outages, including extremes, can be modeled concurrently. (Nonlinear approaches, in theory, allow one to model any number or combination of failure, maintenance, and restoration events, provided there are sufficient data to characterize them.)

Although a number of factors affect the resilience of electricity service, long-duration outages—our focus here—merit special attention. These extremes have an outsized impact on reliability, and omitting them from planning tools carries two fundamental risks. First, when models are not given the full spectrum of outages that degrade utility service, they may underestimate a microgrid's resilience benefits. That is important because a microgrid's ability to add value by improving reliability and resilience is the principal driver of investment in these systems. Second, because investment is required to protect against outages, models risk under-designing microgrids when they do not know about the full spectrum of outages, leading to designs that are reliable on paper but vulnerable in the real world. For example, as the US Department of Defense has documented, not modeling reliability fully has led to microgrid deployments at military installations that do not meet requirements for surviving long-duration outages (Marqusee et al., 2020). Failing to model extremes therefore has two main potential consequences: it leads to fewer investments being made, since, on paper, microgrids appear to have a lesser value of resilience and hence lesser value overall; and, for those investments that are made, deployments evoke a false sense of security, since they appear reliable in the modeling world yet may not stand up to extremes in reality. We therefore expect that efforts to integrate long-duration outages in models would yield larger market potentials for new deployments while also preventing unresilient deployments from reaching final investment decision.

With a growing emphasis on resilience-based planning (Rickerson et al., 2019) (Moreno et al., 2020) (Ratnam et al., 2020) and plausible new waves of investment in resilient infrastructure in the pipeline (Wood Mackenzie, 2021a, 2021b), it is imperative that research efforts address model design challenges related to reliability, resilience, and the effects that long-duration outages have on these; it is also imperative that, with help from stakeholders in industry and government, they have the outage data needed to support these modeling efforts.

## Power outage data and characterization

Electric power outages vary in magnitude and duration, in space, and over time (Larsen et al., 2020) (Dunn et al., 2019). Resilience planning, which seeks to mitigate the effects of high-impact low-probability extremes, should capture this variation as thoroughly as possible. There are at least three approaches for characterizing variation. Ideally, one would adopt a bottom-up approach that uses individual interruptions, their duration, and the number of customers they impact. With sufficient number, individual interruption data can be combined to generate expected values as well as entire probability distributions for the likelihood and duration of interruptions. Although some studies have been given proprietary access to high-resolution data (Kankanala et al., 2014) (Nateghi et al., 2014) (Ji et al., 2016), such detailed data are held by utilities and generally not available. A second approach, with foresight, is to scrub interruption data from public-facing utility webpages in real time until a sufficiently large dataset is collected (Dunn et al., 2019). A third approach—the most accessible and general—is to use averaged interruption data filed annually by electric utilities while making assumptions about the shape of the underlying distribution of interruptions. US electric utilities are required to measure and report the reliability of their service territory using three metrics (U.S. EIA, 2021) (IEEE, 2012):

• System Average Interruption Frequency Index, or SAIFI, which describes how often the average customer experiences a service interruption exceeding five minutes;
• System Average Interruption Duration Index, or SAIDI, which describes for how long the average customer experiences interruptions; and
• Customer Average Interruption Duration Index, or CAIDI, which represents the average time a customer is without power before service is restored.

(A fourth metric, Momentary Average Interruption Frequency Index, or MAIFI, describes the frequency of momentary interruptions, i.e., those lasting less than 5 minutes, and is a complement to SAIFI.) These indices capture the average reliability experience across a utility's service territory each year. They are point values, so using them in probabilistic analysis of resilience requires making assumptions about the wider distribution of interruptions, as we will discuss.

**Figure 1:**
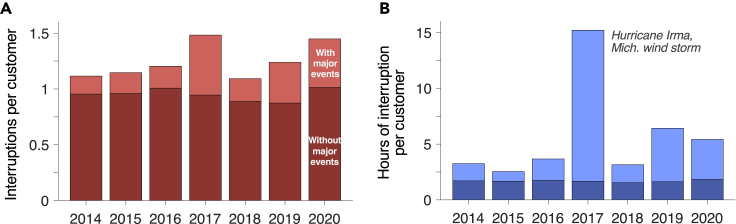
The number and duration of power outages experienced by customers across the nine largest US utilities over 2014–2020, inclusive and exclusive of MEDs (A) SAIFI, in units of interruptions per customer per year. (B) SAIDI, in units of hours of interruption per customer per year. Each of the nine largest utilities (by number of customers; utilities are listed in Figure 2) serves over two million customers. Data are reported annually via Form EIA-861.

Utilities report SAIFI, SAIDI, and CAIDI in two ways: with and without so-called major event days (MEDs; Figure 1). MEDs are days with “major events” that stress the electric grid beyond expected performance and degrade service reliability considerably (IEEE, 2012) (Eto et al., 2017); often they indicate extreme events like severe weather. For example, for the nine largest US utilities, which collectively serve over 32 million customers, major events from 2014 to 2020 were responsible for only 24% of the total number of customer interruptions yet 70% of total service downtime.

Reporting MEDs segments a utility's reliability performance, isolating the effects of MEDs from normal days, and hence provides a formal basis for defining extremes. When indices are reported with and without MEDs, one can characterize the likelihood and duration of two distinct classes of interruption: moderate interruptions (which are the non-MED indices) and long-duration interruptions (which are the difference between non-MED and MED indices). The threshold outage duration that delineates normal and major days is derived statistically for each utility, varies by utility, and varies over time based on past utility performance (Eto et al., 2017).

**Figure 2:**
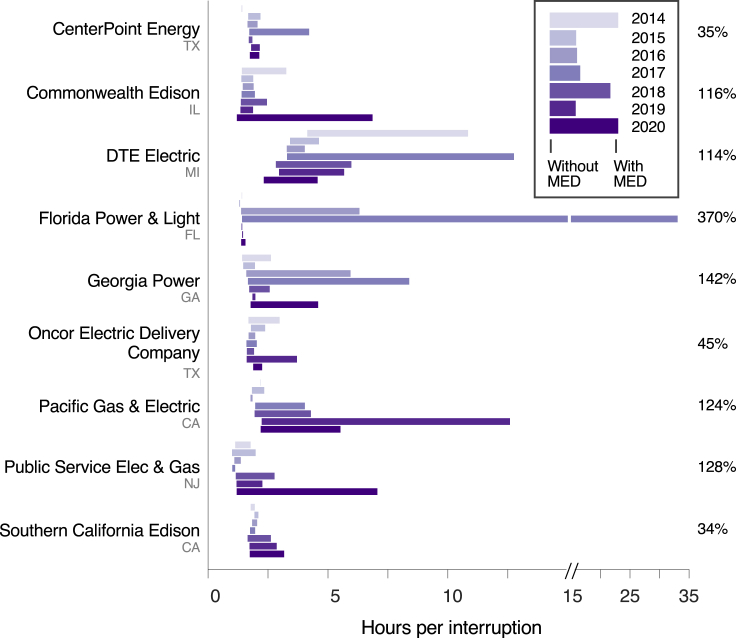
The average duration of interruptions, i.e., CAIDI, experienced by customers across the nine largest US utilities over 2014–2020, inclusive and exclusive of MEDs Indices are annual averages over the utility's service territory. The percentages at right give the mean difference in CAIDI with and without MEDs over 2014–2020 for each utility. Each of these utilities serves over 2 million customers; altogether the data capture the experience of over 32 million customers. In total, electric utilities that collectively serve over 95% of US customers report these data.

Long-duration outages are revealed in the differences between non-MED and MED indices (Figure 2). Major events, which are rare, do not much increase SAIFI. But they can significantly increase average service downtimes (i.e., SAIDI and CAIDI), in several cases more than doubling them, as the percentages in Figure 2 indicate.

**Figure 3:**
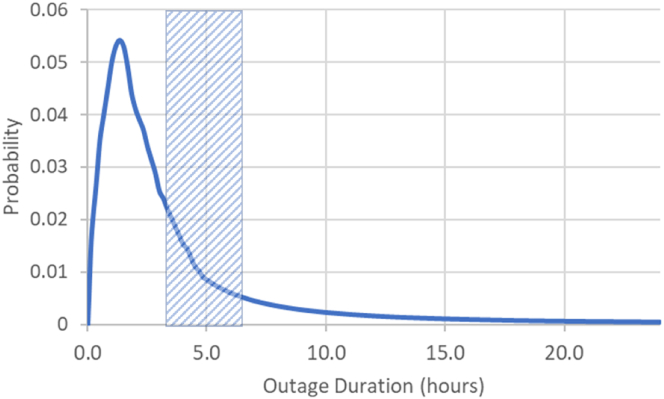
Stylized probability density function (pdf) for the duration of interruptions, i.e., CAIDI, that customers experience The vertical hatched area depicts a range of typical thresholds for outage duration, derived statistically (IEEE, 2012), that delineate MEDs from normal days. Events to the left of the hatch indicate moderate outages on normal days; to the right of the hatch, the tail of the pdf indicates rare but impactful long-duration outages and decays slowly as a power law. Interruption duration pdfs vary by utility and over time.

Utility-reported indices are averages—they capture the reliability experience of the average customer but do not reveal the wider range of interruptions that occur. When the shape of these wider probability density functions (pdf) is not known, it is typical in reliability analysis to assume that event frequency and duration follow standard fits, such as exponential or log-normal distributions (Billinton and Allan, 1996) (Billinton and Allan, 1992). However, real data can reveal other fits. In the pdf for interruption duration (Figure 3), moderate interruptions constitute the body (left of the hatched area in Figure 3) and typically follow a normal or log-normal distribution; however, long-duration interruptions, which constitute the tail, typically follow a power law (Kancherla and Dobson, 2018). For instance, data from several countries suggest that the frequency of large blackouts follows a power-law tail (Dobson et al., 2007). In addition, the size of large-scale power outages, measured as the number of customers *N* impacted by an outage, decays as a power law *N*^*–z*^, where *z* is estimated to be less than 2, typically 1.9 (Carreras et al., 2016). Bulk power transmission restoration times *t* also decay slowly via the power law *t*^*–z*^, where *z* ≈ 1.84 (Kancherla and Dobson, 2018). Because customer interruptions stem from both transmission and distribution outages, it is expected that the duration of interruptions that customers experience similarly follows the power law *t*^*–z*^, where *z* is again less than 2; an initial estimate based on two years of US data (PowerOutage.us, 2021) finds *z* ≈ 1.9.

The power tail implication is significant because power tails, or “fat” tails, decay more slowly than exponential fits, implying that extreme events, although rare, occur with much greater frequency than suggested by often-assumed exponential fits (Kancherla and Dobson, 2018) (Hines et al., 2009). It is therefore crucial that tails are properly represented, since using the lower likelihoods embodied in exponential statistics could lead to infrastructure designs that appear reliable in models but cannot withstand real-world extremes, especially as tails plausibly become fatter as the climate warms.

**Figure 4:**
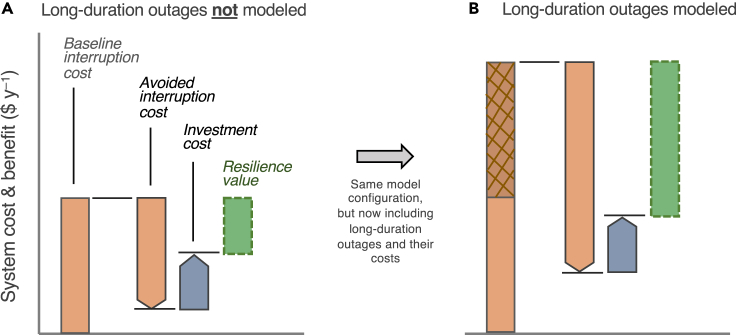
Waterfall chart of the costs and benefits of a resilience investment, showing the interruption cost, investment cost, and value of resilience (A and B) Shown are two model configurations that differ only in the breadth of grid outages input into the model: in (A), only moderate outages are included; in (B), moderate and long-duration outages are included. Interruption costs (leftmost bar) are the economic losses a customer incurs due to outages, which depend on the customer's value of lost load. In (B), the hatched bar indicates additional interruption costs that the model sees once long-duration outages are included. The three rightmost bars illustrate how costs shift following investment. The dashed (rightmost) bars indicate the resilience value (i.e., the difference between the costs of investment and costs of interruption that the investment avoids) determined by the model. Investment costs in the two cases could be very different (for example, if energy-limited resources are used) or largely similar (for example, if uninterruptible fuel supplies, like natural gas, are available).

Resilience investments, such as microgrids, shuffle system costs: new infrastructure carries upfront capital costs, but over time it avoids losses caused by interruptions. It generates a net financial benefit—a resilience value—when avoided interruption costs exceed investment costs (Figure 4A). Long-duration outages, embodied in the slow decay of the outage duration pdf in Figure 3, can profoundly impact this shuffling of costs and benefits, since the resilience value that can be banked depends on the magnitude of interruption costs that can be avoided. Including long-duration outages in models adds new baseline interruption costs and, with them, the opportunity to avoid such costs through investment (Figure 4B). In modeling that omits long-duration outages, investments have smaller resilience values; and in the most acute cases, resilience values can appear negative, suggesting that investment costs would eclipse avoided interruption costs and that investment would be, from a resilience standpoint, uneconomic.

Including long-duration outages in models can fundamentally reshape the cost-benefit calculus, making apparently uneconomic investments appear, correctly, economic. Moreover, the additional investment needed to protect against extreme multi-day outages, relative to protecting against moderate multi-hour outages, can be modest, such as when uninterruptible natural gas supply is available (Bohman et al., 2021). As Figure 4 illustrates, including long-duration outages can lead to moderate shifts in investment but large increases in resilience value. In some circumstances, resilience benefits can grow faster than the costs of protecting against long-duration outages. This can happen when the outage duration pdf tail decays more slowly than t^−2^, as data suggest it does, and cumulative economic losses from outages grow at least linearly with outage duration. The potential losses from outages (and hence resilience benefits) are, in theory, unbounded. In practice, outages have finite duration, and it is the full set of outages that customers experience, along with customers' value of lost load, that shapes whether resilience benefits outstrip new costs of investment.

## Integrating power outages in planning tools

For resilience-based planning and modeling, the first step is to characterize distinct types of disruptions, such as for long-duration, moderate, and momentary outages, by generating probability distributions for their likelihood and duration. The second step is to design planning tools that can actually assimilate these distributions. We describe two broad design approaches—linear and nonlinear—and their merits and demerits.

Microgrid planning tools, which determine the set of DER investments that minimize a microgrid's total life cycle cost (thereby maximizing its financial return), have been under development since the mid-1990s (Lilienthal et al., 1995), spearheaded by US National Laboratories (Marnay et al., 2001) (Feng et al., 2018) (Cutler et al., 2017) and later picked up by industry (Pecenak et al., 2019). A general, stylized model formulation is given in Equation 1, where *C* denotes cost, *x* the set of DER investments, and *y* DER operation; and where the investment cost comprises that of distinct DERs and related gear, the operating cost includes electricity, fuel, maintenance, and emissions components, and interruption costs are the economic losses from power outages. (For a complete formulation see, for example, Cutler et al. (2017).

(Equation 1) minimize *C*_total_*(x,y)* := *C*_investment(_*(x)* + *C*_operation_*(x,y)* + *C*_interruption_*(x,y)*subject to physical constraints (energy flow, DER capabilities and limitations, fuel availability)financial constraintsemissions constraintsreliability and resilience constraints

Although model development has been directed chiefly toward modeling energy flows, supplies, and demand (and not reliability per se), recent development has pivoted toward issues of resilience. For example, recent work has looked at probabilistic resilience calculations (Hanna et al., 2018; Dong et al., 2018; Anderson and Mishra, 2021), resilience-based business cases for microgrids (Hanna et al., 2017), household requirements for resilience (Bohman et al., 2021), how better resilience can lower insurance premiums (Anderson et al., 2018), and how to model time-varying losses from outages (Anderson et al., 2021). One study modeled intentional utility power shutoffs in California aimed at preventing wildfires—a particular instance of long-duration outage with a distinctive duration fingerprint (Hanna, 2021). Yet development has not tackled long-duration outages generally, their fat-tailed distributions, or the effects they have on optimal DER selection in microgrids and on system reliability and resilience.

The central modeling challenge with reliability is the structural model complexity that outages introduce, since reliability is a nonlinear function of investment (Billinton and Allan, 1996). Reliability, a probabilistic measure of how often electricity supply meets demand, follows the binomial distribution *R(N,x)* ∝ *A*^*x*^*(1–A)*^*N–x*^, where *N* is the number of thermal generators, of which *x* is the number that must operate concurrently for the system to function and *A* is the generator's availability (Billinton and Allan, 1992). Nonlinear complexities are thus present even for simple power systems, such as those comprising only a set of *N* thermal generators. Yet microgrids can be complex, host to a diverse array of DERs. Other events, like DER failures, also degrade system reliability and further complexify calculations of reliability. Assuming perfect DER reliability, as studies often do, leads to overestimates of system reliability, in particular during long-duration outages when DERs serve as the last line of defense against outage (Marqusee et al., 2021a, 2021b). In addition, some DERs are energy-limited, others depend on weather, and many must be routinely removed from service for maintenance. Backup generators can fail to start (Marqusee and Jenket, 2020) and switchgear can fail to isolate critical loads. Meanwhile, extreme events can take down multiple DERs simultaneously in common-cause failures (Mosleh, 1991). All of these phenomena planning models should, ideally, capture.

**Figure 5:**
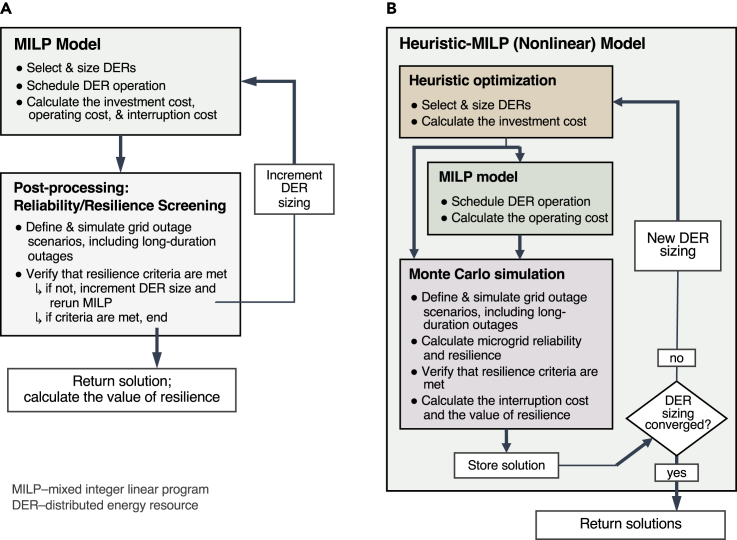
Microgrid planning tool architecture for linear and nonlinear modeling approaches for integrating long-duration outages (A) Linear models accept a single set of pre-defined grid outages in the core MILP module and represent the interruption cost linearly as the product of value of lost load and unsupplied energy. Post-processing routines can be added to analyze other outages and resilience in greater detail and, with iteration, used to inform new DER investment. (B) Nonlinear models delegate cost calculations to various subroutines, which can include conventional linear models and MCS reliability evaluation algorithms. When integrating MCS, nonlinear models simulate and capture thousands of combinations of DER and grid failure scenarios before calculating system resilience and interruption cost using customer damage functions.

There are, broadly, two modeling approaches that can be taken to address these challenges while also integrating long-duration outages (Figure 5). The first is to stick with conventional linear models. As the basis for existing planning tools, this approach would retain substantial prior model development and know-how. Linear models do well with the costs and benefits associated with energy flows, which are easy to linearize. However, to treat outages and reliability they require a number of simplifying linearizations and are commonly parameterized with assumptions of perfect DER reliability or a single prescribed (e.g., average) grid outage frequency and duration (Marnay et al., 2008) (Simpkins et al., 2014). It is known that these simplifications introduce inaccuracies in model results for optimal investment and resilience benefits (Marqusee et al., 2021a, 2021b). More detailed reliability calculations can be made aside linear models, for example, via pre-processing (Laws et al., 2018) or post-processing (Mishra et al., 2021) routines that permit simulation of a wider array of moderate and long-duration outages, parameterized with full pdfs, or that ensure resilience constraints are met. Nevertheless, because these finer resilience calculations are not integrated within the optimization framework (Figure 5A), they do not inform the inherent trade-off between investment costs and resilience benefits; thus it is not clear whether these coupled models produce optimal investment choices.

The second approach prioritizes reliability above all else by maintaining the fidelity of the nonlinear reliability term. Instead of linear models, this approach uses heuristic optimization methods that can integrate nonlinear reliability calculations, even entire simulation subroutines, alongside conventional linear programs (Figure 5B) (Hanna et al., 2019). Indeed, with simulation-based methods for calculating reliability, notably sequential Monte Carlo simulation (MCS) (Billinton and Allan, 1996), modelers can characterize any event known to impair reliability—DER failures, maintenance downtime, common cause failures, etc.—provided sufficient data are available to characterize it. That includes long-duration outages, defined by pdfs for likelihood and duration (as discussed in the previous section), as well as analogous distributions for momentary and sustained outages. Moreover, this diversity of outages directly informs DER investment, since investment choices, operation decisions, and reliability calculations are housed in a single, integrated optimization framework. The trade-off is greater model complexity and computational complexity, but the advantages include more comprehensive and accurate estimates of reliability and resilience benefits.

A comprehensive comparison of these two approaches, to our knowledge, has not been undertaken. The effects of using one approach or another on optimal system design, costs, and benefits are therefore not well established. What limited evidence does exist suggests that differences may be very large. One study, adopting both approaches separately, found 50% difference in investment for key DERs like gas generators, leading to nearly 50% difference in perceived cost-effectiveness and, remarkably, 600% difference in system reliability (Hanna et al., 2019). In other words, a simplified linear approach for reliability yielded optimal microgrid designs that were 600% less reliable than those found by models that maintained the reliability nonlinearity. These results underscore the importance of properly characterizing reliability in models, but further research is needed to draw general conclusions about these large differences.

## Future needs for data and modeling

Extremes are important to examine because they are known to severely impact society and human welfare (Taleb, 2010). In different ways, they pose challenges for grid regulators responsible for maintaining resource adequacy as well as for individual customers and firms seeking higher levels of electric reliability than those provided by local utility service. Yet long-duration outages are usually not well characterized in the tools used to plan resilient grid infrastructure.

We have discussed how certain barriers, like lack of access to comprehensive power outage datasets and ill-suited tools, make it difficult to analyze complex interactions between long-duration outages and resilience. Approaches for characterizing outages are possible, for example, by using annual average reliability indices reported by utilities while making assumptions about the wider spread of interruptions. Fundamentally, however, improving estimates of the economics and resilience of grid investments will require wider access to the granular interruption data that underlie rare events and fat tails.

We conclude by highlighting three ongoing challenges related to data and modeling of resilient decentralized energy systems that could be emphasized in future efforts to improve grid planning tools. These challenges are large but tractable and may require collaboration among researchers, utilities, industry, and government. The consequences of not improving planning tools, ahead of plausible waves of investment in new resilient infrastructure, include potentially long-lived investments that prove unresilient, uneconomic, or both.

### Historical outage data and future projections

Customer interruption pdfs can have power law tails that define rare events. Characterizing these pdfs requires large datasets on individual interruptions that span many years. However, access to interruption data is limited to the average metrics reported annually by utilities via Form EIA-861 (Figures 1 and 2) or records maintained by the North American Electric Reliability Corporation on the size and duration of large transmission-level outages (NERC, 2021). Neither are sufficient to characterize customer interruption pdfs. Only in select cases have utilities published comprehensive datasets on customer interruptions (Chowdhury and Doostan, 2017) (Jaech et al., 2019) (Mukherjee et al., 2018), but pdfs and power law tails are difficult to characterize with small data sets—annual average outage duration, for example, can vary substantially year to year (Kancherla and Dobson, 2018). Utilities maintain large interruption datasets in outage management systems, but these have been mostly untapped by researchers. With appropriate safeguards to maintain anonymity and protect sensitive information, access to these data repositories would be hugely beneficial to researchers. Utilities could likewise benefit through more sharing; even only a handful of utilities engaging in sharing would open up substantial opportunities for new resilience research, which could eventually diffuse into utility integrated resource and other procurement planning.

Yet historical data alone are insufficient to characterize interruptions. New resilient infrastructure is expected to last decades, yet extreme weather, a non-stationary process, is shifting quickly owing to global warming (Larsen et al., 2020). Advances in climate research are making it possible to predict how extremes may shift in the future; it is equally vital to translate this improving ability to predict shifting baselines for extreme weather into shifting baselines for power outages and other energy system impacts (Brockway and Dunn, 2020) (Perera et al., 2020). Also needed is an understanding of how various reliability markers are or are not affected by shifting extremes. It is plausible, for example, that more extremes will lead to many more long-duration outages while not much affecting rates of short-lived outages.

### Value of resilience during long-duration outages

Long-duration outages have outsized economic impact on modern societies. Calculating the resilience value of new infrastructure—i.e., the margin between new costs of investment and avoided costs of interruption—requires modeling how well infrastructure protects against outages. It also requires knowing how outages lead to economic losses, a relationship that is nonlinear and varies by customer, outage type, and outage duration (Thomas and Wilhelm, 2015) (Sullivan et al., 2018). Although substantial work has been done to characterize such values of lost load (VoLL), almost all estimates are restricted to shorter duration outages of less than 12 h (Sullivan et al., 2015) (Anderson, 2020). Moreover, planning tools often treat VoLL as a scalar dollar-per-kWh value when in actuality VoLL is known to vary with outage duration (Thomas and Wilhelm, 2015) and a number of other factors. What is needed are estimates for VoLL over multi-day outages (Larsen et al., 2019) (Baik et al., 2020), which may require new approaches since most customers have limited experience with long-duration outages (Zamuda et al., 2019) (Baik et al., 2020). Establishing VoLL is particularly difficult when other infrastructures are simultaneously damaged and requires intimate knowledge of the correlations between power outages and other infrastructure damages.

### Power component reliability

In microgrids, bulk grid reliability is the chief determinant of overall service reliability. DER reliabilities are second in importance and, although they have modest impact on overall system reliability during short-duration outages, they can have a material effect during long-duration outages, since a component's likelihood of failing increases with its time in service. However, the last major efforts to collect power component reliability data were arranged over 20 years ago. Although those efforts yielded a huge resource—the Institute of Electrical and Electronics Engineers (IEEE's) Standard 3006.8, which enumerates component reliability data and recommends best practices (IEEE, 2018)—the resulting data are now outdated. What is lacking and needed are data on many modern power components, such as lithium-ion batteries, fuel cells, renewable DERs, and modern inverters and generators. The existing IEEE resource holds great value as the chief source of data, but without updates to include modern equipment we are left guessing at the reliability of our evolving power system.
